# Unraveling the Influence of Social, Economic, and Demographic Factors in Texas on Breast Cancer Survival

**DOI:** 10.21203/rs.3.rs-4535192/v1

**Published:** 2024-06-25

**Authors:** Sidketa Ida Fofana, Tamer Oraby, Everado Cobos, Manish K. Tripathi

**Affiliations:** University of Texas Rio Grande Valley; University of Texas Rio Grande Valley; ISU, University of Texas Rio Grande Valley; ISU, University of Texas Rio Grande Valley

**Keywords:** Breast Cancer, Survival Analysis, Cox Proportional Hazard, Racial Equity

## Abstract

**Background:**

According to the Centers for Disease Control (CDC), breast cancer is the second most common cancer among women in the United States. Affected people are financially challenged due to the high out-of-pocket cost of breast cancer treatment, as it is the most expensive treatment. Using a 16-year cohort study of breast cancer survival data in Texas, we investigate the factors that might explain why some breast cancer patients live longer than others.

**Methods:**

Performing a survival analysis consisting of the log-rank test, a survival time regression, and Cox proportional hazards regression, we explore the breast cancer survivors’ specific attributes to identify the main determinants of survival time.

**Results:**

Analyses show that the factors: stage, grade, primary site of the cancer, number of cancers each patient has, histology of the cancer, age, race, and income are among the main variables that enlighten why some breast cancer survivors live much longer than others. For instance, compared to White non-Hispanics, Black non-Hispanics have a shorter length of survival with a hazard ratio of (1.282). The best prognostic for White non-Hispanics, Hispanics (all races), and Black non-Hispanics is a woman aged between 40 to 49 years old, diagnosed with localized stage and grade one with Axillary tail of breast as a primary site with only one cancer and with a household income of 75,000.00 and over.

**Conclusion:**

Policymakers should promote early diagnosis and screening and better assist the older and the poor to improve the survival time for breast cancer patients.

## Introduction

According to the American Cancer Society (ACS) 2024 statistics and Centers for Disease Control and Prevention (CDC), breast cancer is the second most common cancer after skin cancer among women in the United States across all races and ethnicities. In fact, in 2024, 313,510 women will be diagnosed with breast cancer, and 42,780 women in the United States are expected to die from breast cancer[1, 2]. In the United States of America, one in every eight women will be diagnosed with invasive breast cancer during her lifetime, according to the National Cancer Institute. Breast cancer national expenditures in the USA were estimated at $29.8 billion in 2020 [3]. Furthermore, [4], who compared the treatment costs for breast cancer by tumor stage using a sample size of 8,360 women, found that the average costs per patient allowed by the insurance company in the year after diagnosis were $60,637 for stage 0, $82,121 for stage I and II, $129,387 for stage III and $134,682 for stage IV. Therefore, Breast cancer is a burden to taxpayers; it is a public health issue, and breast cancer mortality is associated with huge productivity loss.

In this study, we focus on the state of Texas. Breast cancer is the most diagnosed cancer in Texas women and the second leading cause of cancer death in Texan women[5]. In 2022, DSHS estimated that 19,921 women were diagnosed with breast cancer in Texas, with a death toll of 3,415 women.

Cancer is a disease in which cells in the body grow out of control. When cancer starts in the breast, it is called breast cancer. It can spread outside the breast through blood vessels and lymph vessels. When breast cancer spreads to other parts of the body, it is said to have metastasized. The most common kinds of breast cancer are invasive ductal carcinoma and Invasive lobular carcinoma (CDC). The seed and soil theory in metastasis, proposed by Stephen Paget in 1889, likens the spread of cancer cells to the seeding of fertile land [6]. Metastatic tropisms refer to cancer cells’ preferences for specific organs or tissues when metastasizing. Various factors contribute to these preferences, including the relations between the cancer cells and the microenvironment of the target organ. For instance, breast cancer cells tend to metastasize to the brain, bones, and lungs [7, 8], while lung cancer cells often spread to the brain or other lung tissues [9]. Research studies have provided insights into the molecular mechanisms underlying metastatic tropisms and the interactions between circulating tumor cells and the microenvironment of target organs [10–12]. Understanding the intricate details of metastatic tropisms and the interaction between malignant cells and the microenvironment of target organs remains an active area of research in oncology. These insights hold promise for developing targeted therapies to disrupt the mechanisms that drive metastasis, potentially improving outcomes for cancer patients.

We use the 16-year cohort study of breast cancer survival data in Texas from [13] to identify the health and socio-economic disparities in breast cancer survival that might explain why some breast cancer patients live longer than others. Knowing those factors makes it easy for us to identify the riskiest cancer survivors. We will be able to make effective and resourceful recommendations to help new patients have a much longer and better life and reduce breast cancer burden to society. We include only people who were diagnosed with malignant breast cancer in 2004 and 2005 by the SEER and were followed up to 2020. The SEER recorded 25,184 cases of such description.

We performed 2-step survival analyses on the entire data set. The first step consisted of running a log-rank test and a survival time regression. The second step consisted of running Cox proportional hazards regression. All those different techniques help better understand the main drivers of breast cancer survival and the disparities that may exist between those variables.

The rest of the paper is organized as follows: a background and a literature review, then a data description and characteristics, statistic models and results, and finally, a conclusion.

## Background

According to the Centers for Disease Control and Prevention, cancer is a disease in which cells in the body grow out of control. When cancer starts in the breast, it is called breast cancer. It can spread outside the breast through blood vessels and lymph vessels. When breast cancer spreads to other parts of the body, it is said to have metastasized. The most common kinds of breast cancer are invasive ductal carcinoma and Invasive lobular carcinoma.

The main risk factors that lead to breast cancer include old age, genetic mutations, early menstrual period, late or no pregnancy, starting menopause after age 55, not being physically active, being overweight or obese after menopause, having dense breast, using a combination of hormonal therapy, taking oral contraception’s, family history of breast cancer, drinking alcohol, smoking, cigarettes and women from high socio-economic status [14–16]. It is, therefore, challenging to prevent breast cancer.

Yet, a mammogram is a screening tool that physicians can use to find breast cancer early, sometimes up to three years before it manifests the first physical symptoms. A mammogram is an X-ray picture of the breast. Mammography can also be used as a diagnostic tool to confirm the presence of breast cancer. However, mammography is not perfect since errors in diagnostics can happen [17]. A mammography can make false positive results, primarily for young women and women with dense breast size. It can also find false negative results that can delay treatment and lead to a false impression of security for affected women. Finally, it is evident that the risk of harm from radiation exposure from mammography is low, but repeated X-rays have the potential to cause cancer [17]. Recently, there has been a new type of mammogram for breast cancer detection tomosynthesis called 3D mammogram. In January 2018, Texas required all insurance companies to cover 3D mammograms for Texas patients. There is a need to continue investing in research for better screening and diagnostic tools in the future, which will lead to early detection and reduce breast cancer mortality.

Since breast cancer can be a recurrent or chronic disease, the main goal for survivors is to maximize their survival time. Therefore, it is essential to understand why some survivors live longer than others. Besides, cancer mortality is associated with a huge productivity loss, as shown by [18], which studied productivity costs of cancer mortality in the United States: 2000–2020. They developed models using the human capital approach, which relies on earnings as a measure of productivity, to estimate the value of productivity loss due to cancer mortality. They developed models using the human capital approach, which relies on earnings as a measure of productivity, to estimate the value of productivity lost due to cancer mortality. The annual productivity cost from cancer mortality in the base model was approximately $115.8 billion in 2000; the projected value was $147.6 billion for 2020. Including imputed earnings loss due to caregiving and household activity increased the base model total productivity cost to $232.4 billion in 2000 and $308 billion in 2020. Their paper is helpful to our analysis, as it shows productivity costs and all related costs due to cancer mortality. Therefore, it is important to understand that the longer cancer survivors live, the more it helps minimize the loss of productivity.

In addition, there are costs associated with breast cancer survivors. According to [19], productivity costs related to breast cancer among survivors aged 18–44. They find that, per capita, younger women with breast cancer had annual losses of $2,293 (95% CI=$1,069, $3,518) from missed work and $442 (95% CI=$161, $723) from lack of productivity at home. Total annual breast cancer–associated productivity costs for younger women were $344 million (95% CI=$154 million, $535 million). Older women with breast cancer had lower per capita work loss productivity costs of $1,407 (95% CI=$899, $1,915) but higher total work loss productivity costs estimated at $1,072 million (95% CI=$685 million, $1,460 million) than younger women. As a result, the benefit of survival exceeds the cost of death in terms of finance, economic, and labor productivity.

We also need to mention that most of those women are someone daughters, mothers, or grandmothers, which implies emotional and social loss as well. In the case of the premature death of a young mother, for instance, the hiring of a caregiver to tend to her children can be challenging and costly.

In the literature, personal characteristics such as race, age, lifestyle, physical activities, diet, treatment, and stage of detection of cancer, such as mammography, radiotherapy, or chemotherapy, can explain the survival time for breast cancer patients.

### 2.1 Breast cancer survival and race:

An increasing disparity in breast cancer mortality from 1979 to 2010 for African American women aged 20 to 49 has been studied [20]. They find that disease-specific mortality rates declined over time for selected conditions. Still, mortality rates were persistently higher for black women for breast cancer, cervical cancer, colorectal cancer, ischemic heart disease, and stroke. The mortality rate ratio increased for breast cancer across the study’s period. The annual mortality rate ratio for black women compared to white women was 1.36 in 1979 compared to 2.00 in 2010.

Furthermore, a racial difference in breast cancer mortality by stage at diagnosis since mammography became available has been assessed [21]. They calculated adjusted odds of distant (versus local or regional) tumors for 14,3249 white and 13,571 black women aged 50 to 69 years diagnosed with breast cancer between 1982 and 2007. In conclusion, in the mammography era, racial disparities remain in the stage at diagnosis.

The ability of recognized prognostic factors for breast cancer to account for the observed poorer survivorship in blacks compared to their white counterparts has been examined [22]. Multivariable survival models were used to estimate the hazard ratio (relative risk of mortality) for blacks compared to whites, adjusting for various combinations of potential explanatory factors. They found that approximately 75% of the racial difference in survival was explained by the prognostic factors studied. Socio-demographic variables appeared to act mainly through racial differences in the stage at diagnosis, which may be amenable to change through improved access to and use of screening for black women. Therefore, early screening is vital to improve the survival time of breast cancer patients. Earlier stage of diagnosis and improved survival among Medicare Health Maintenance Organizations (HMO)patients with breast cancer were correlated [23]. They used a linkage of two national databases, the Medicare database from the Centers for Medicare and Medicaid Services (CMS) and the National Cancer Institute’s (NCI) Surveillance, Epidemiology, and End Results (SEER) program database to evaluate differences in demographic data, stage at diagnosis, and survival in patients with breast cancers over the period 1985–2001. Their results have indicated that Medicare patients enrolled in HMOs were diagnosed earlier than fee-for-services (FFS) patients. HMO patients diagnosed with breast cancer had improved survival rates, and these differences remained even after controlling for potential confounders. Specifically, breast cancer patients enrolled in HMOs had a 9% increased probability of survival (hazard ratio [HR] 0.91, 95% confidence interval [CI] 0.88–0.93) than their counterparts enrolled in FFS. These findings persisted even when patients had a cancer diagnosis before their breast cancer. These findings show that improved survivorship among breast cancer patients in HMOs compared to FFS is likely due to a combination of factors, including but not limited to earlier stage at the time of diagnosis, which reinforced the idea that early diagnosis is key to improving survival time for breast cancer patients.

Investigating reasons for ethnic inequalities in breast cancer survival in New Zealand found that inequalities persisted after adjustment for subtype variables (ER/PR/HER2) while adjusting for access to care variables (extent/size) eliminated the ethnic inequalities in excess mortality [24]. They conclude that ethnic disparities in breast cancer survival in New Zealand can be attributed to deprivation and differential access to health care rather than differences in breast cancer subtypes. I then control for race and ethnicity in my study to see if there is any disparity in survival time due to race, given that Texas is diverse regarding race and ethnicity.

### 2.2 Age at diagnosis and survival of breast cancer

Age at diagnosis is an important factor for breast cancer survivorship [25], the relation between age at diagnosis and relative survival in 57,068 women in Sweden in whom breast cancer was diagnosed in 1960 to 1978. Their findings suggest that women who were 45 to 49 years old had the best prognosis, with a relative survival exceeding that of the youngest patients. They also found that the relative survival declined markedly after the age of 49, mainly in women aged 50 to 59 and women older than 75. They conclude that the long-term annual mortality rate due to breast cancer approached 1 to 2 percent at the premenopausal ages but exceeded 5 percent throughout the period of observation in the oldest age group.

Additionally, changes in physical and psychosocial function before and after breast cancer by age at diagnosis were observed [26]. From a large sample size of 122,969 women from the Nurses’ Health Study (NHS) and NHS 2, ages 29 to 71 years, who responded to pre- and post-functional status assessments. Among them, 1,082 women were diagnosed with breast cancer between 1992 and 1997. Functional status was measured using the Medical Outcomes Study Short Form 36 (SF-36). Mean change in health-related quality of life (HRQoL) scores were computed across categories representing the combination of incident breast cancer (yes or no) and age at diagnosis (40, 41 to 64, or 65 years). They conclude that compared to women 40 years without breast cancer, women with breast cancer experienced significant functional declines. Young (age 40) women who developed breast cancer experienced the most important relative declines in HRQoL (as compared to middle-aged and older women) in multiple domains, including physical roles (18.8v 11.5and 7.5points, respectively), bodily pain (9.0v 2.7 and 2.7 points), social functioning (11.3 v 4.3 and 4.4 points), and mental health (3.1 v 0.0 and 0.4 points). Much of the decline in HRQoL among elderly (age 65) women with breast cancer was age-related. In my study, we will then see if age at diagnosis is still an important factor in survival time for breast cancer patients in Texas.

### 2.3 Comorbidity and Breast Cancer Survival

The existence of comorbidities, such as diabetes and asthma, is a factor in early death due to breast cancer. Racial differences in the effects of comorbidity on breast cancer-specific survival have been studied [27]. They are using a retrospective cohort study of 68,090 women 66 + years old who were diagnosed with stage I–III breast cancer in the United States from 1994 to 2004. Their findings suggest that diabetes without complications was associated with a significantly increased hazard for breast cancer-specific death among white breast cancer patients, and aggressive tumor characteristics explained some of this effect. Also, hypertension was associated with an earlier stage of breast cancer diagnosis for both black and 2.3.1 white women (p < 0.01). For black women, hypertension was also associated with a less aggressive tumor grade (p = 0.02), and for white women, hypertension was associated with a less aggressive hormone receptor status (p < 0.01).

Furthermore, obesity, smoking and alcohol, and the existence of comorbidity are also negatively influencing the survival time for breast cancer patients.

#### 2.3.1 Obesity and Breast Cancer

Most recent studies agree that obesity is a determinant for both getting breast cancer and surviving long from it. Obesity, physical activity, and breast cancer survival among older breast cancer survivors in the Cancer Prevention Study-II Nutrition Cohort for 5254 between 1992 and 2013 have been reported [28]. They conclude that Higher BMI, pre- or post-diagnosis, was associated with a higher risk of breast cancer-specific mortality in older patients, independent of comorbidity and stage at diagnosis.

Likewise, a study on the relationship between obesity and quality of life (QOL) among Hispanic and non-Hispanic white breast cancer survivors and population-based controls from the Long-term Quality of Life Study, a 12- to 15-year follow-up study of breast cancer cases/survivors and controls from New Mexico (n = 451, has been performed [29]. They used Body Mass Index (BMI) to measure obesity, and follow-up interviews were modeled with composite scores for physical and mental health from the SF-36 Quality of Life Survey. Interaction between ethnicity and BMI and change in BMI were evaluated. All models were adjusted for age, ethnicity, Charlson Index, depression, fatigue, and physical activity. They found that baseline obesity (b = −6.58, p = 0.04) was significantly associated with decreased mental health among survivors but not among controls. Obesity at baseline and follow-up was significantly associated with decreased physical health among survivors (baseline b = −10.51, p = 0.004; follow-up b = −7.16, p = 0.02) and controls (baseline b = −11.07, p\0.001; follow-up b = −5.18, p = 0.04). No significant interactions between ethnicity and BMI were observed.

#### 2.3.2 Stage and grade at diagnostic and breast cancer survival

The stage and the grade of the cancer at the time of diagnosis play a major role in the length of survivorship and the treatment the patient is expected to receive. Staging is used to evaluate the size of a tumor, whether it has spread, and how far it has spread. Understanding the stage of the cancer helps doctors predict the likely outcome and lay out a treatment plan for each patient. Since we only focus on malignant cancer patients at diagnosis, we will use the localized, regional, distant, and un-staged stages. The National Cancer Institute defined: Localized stage as cancer that is limited to the place where it started, with no sign that it has spread. Regional stage as cancer that has spread to nearby lymph nodes, tissues, or organs. Distant stage as cancer that has spread to distant parts of the body. An unknown stage is when there is insufficient information to figure out the stage. Therefore, the stage of diagnosis is key for survival time. Some studies have confirmed this. According to[30] women diagnosed with breast cancer between 2012 and 2018, the rate of survival from breast cancer by stage after 5 years from diagnosis is the following: 99% for localized stage, 86% for regional stage, and then decrease dramatically for distant stage to only 30%. We need to diagnose the tumor at an early stage if we aim to improve the survival time for breast cancer patients. I control for stage at diagnostic in this study.

Besides the stage of the tumor, the grade of the tumor is an important factor for both survival and treatment options.

The grade of a tumor indicates what the cells look like and gives an idea of how quickly the cancer may grow and spread. Tumors are graded between 1 and 4. Grade 1: the cancer cells look small and uniform like normal cells and are usually slow-growing compared to other grades of breast cancer. Grade 2: The cancer cells are slightly bigger than normal cells, varying in shape and growing faster than normal cells. Grade 3 and 4: The cancer cells look different from normal cells and are usually faster growing than normal cells.

#### 2.3.3 Neighborhood poverty level and household median income and breast cancer survival

Access to quality care is crucial for breast cancer prognostics. Some costs are associated with taking good care of survivors, such as the cost of healthy food needed to maintain physical activity, such as going to the gym.

As a result, financial status plays an important role, and to account for it, I control for neighborhood poverty level based on the census tract of diagnosis address and patient household median income. In fact, from the literature, the neighborhood poverty level where the survivors live and their length of survival time correlate. [31]studied 3 million tumors diagnosed between 2005 and 2009 from 16 states plus Los Angeles, and they were assigned 1 of 4 groupings based on the poverty rate of the residential census tract at the time of diagnosis. The sex-specific risk ratio of the highest-to-lowest poverty category was measured using Poisson regression, adjusting for age and race for 39 cancer sites. They find a negligible relationship between the local poverty rate and cancer incidence overall. However, 32 of 39 individual cancer sites show such an association, with 14 sites associated with higher poverty and 18 sites associated with lower poverty. This includes 19 sites with more substantial evidence of a relationship as indicated by a monotonic increase or decrease across all 4 poverty categories.

I need to mention that the breast cancer incidence rate is higher among women with high standards of living due to their lifestyles, such as getting married at an older age or not getting married at all, having a baby after 30 years old, and not breastfeeding. Perhaps this high incidence can be explained by Charles E Phelps hypothesis of “life in a fast Lane”. This hypothesis suggests that an increase in people’s incomes comes at the expense of health-hazardous industrial processes, causing a decline in health status.

We also looked at where the survivors live, such as metropolitan, urban, or rural areas, since that can impact the types of care the survivors receive. For instance, there are more cancer hospitals in big cities than rural areas and healthier food stores in cities than in rural areas. All these factors can have a significant impact on breast cancer patients’ prognostics.

## Data acquisition, characteristics, and analysis methods

In this retrospective cohort study in Texas, the data for breast cancer cases was provided by the Surveillance, Epidemiology, and End Results (SEER) Program (www.seer.cancer.gov) SEER*Stat Database: Incidence - SEER Research Plus Limited-Field Data, 22 Registries, Nov 2022 Sub (2000–2020) - Linked to County Attributes - Time Dependent (1990–2021) Income/Rurality, 1969–2021 Counties, National Cancer Institute, DCCPS, Surveillance Research Program, released April 2023, based on the November 2022 submission. The study has been determined ‘Exempt’ under the basic HHS Policy for Protection of Human Research Subjects, 45 CFR 46.104(d) with IRB-24–0193 at UTRGV.

## Results

We included only the diagnosis of breast cancer patients recorded by the SEER Program in 2004 and 2005 and were followed up to 2020. In Texas, the SEER program recorded 17,033 cases of breast cancer in white non-Hispanic patients and 2,895 cases of breast cancer in black non-Hispanic patients diagnosed, 4,690 Hispanics, and 566 others. In this data, 99.24% are patients. We then use the length of survival in years for each patient. We perform 2 types of survival analyses. First, we use a survival analysis on length of survival to deeply understand the correlation between survival length and some variables such as race, age, stage, grade, the primary site of cancer, the total number of malignant cancers for each patient, and median household income of the survivors.

Summaries of the variables of the breast cancer patients who were diagnosed in Texas in 2004 and 2005 are given in Table 1.

From Table 1, we notice that White non-Hispanics are much older (23.01% are 75 and older) than Black non-Hispanics (14.72% are 75 and older), than Hispanics (12.84% are 75 and older), and others are much younger since only 5.30% of them are 75 and older.

Based on grade, White non-Hispanics were diagnosed with early grade (50.44% were diagnosed with grade 1 and 2) than others (42.94% were diagnosed with grade 1 and 2), then Hispanics (40.89% were diagnosed with grade 1 and 2) and then Black (33.88% are diagnosed with grade 1 and 2).

Furthermore, White non-Hispanics were diagnosed with early-stage (55.36% were diagnosed with localized stage) compared to Black non-Hispanics (43.07% were diagnosed with localized stage), Hispanics (45.42% were diagnosed with localized stage) and others (50.18% were diagnosed with localized stage).

Based on the median household income, we can observe that 27.99% of White non-Hispanics were from households with a median income of $75,000.00 or over, compared to 19.24% of Black non-Hispanics, 12.05% of Hispanics, and 35.51% of others. Those income discrepancies may have also played a role in the survival length disparities.

We performed a Chi-square test to examine if there are associations between the survivors’ race and the other variables such as stage, grade, age, chemotherapy treatment, number of cancers, median households’ income, and rural-urban continuum code. The results of the Chi-square tests are given in Table 2.

Based on the Chi-Square test, all variables are associated with race.

We performed a log-rank test (*aka* Mantel-Cox test), which compares the distributions of two samples. It is a nonparametric test that is appropriate in examining if the probability of events of two or more groups over time is different (or, in other words, if two or more groups have different hazard functions) when the data is right censored as in the case of our study.

**Figure 1:**
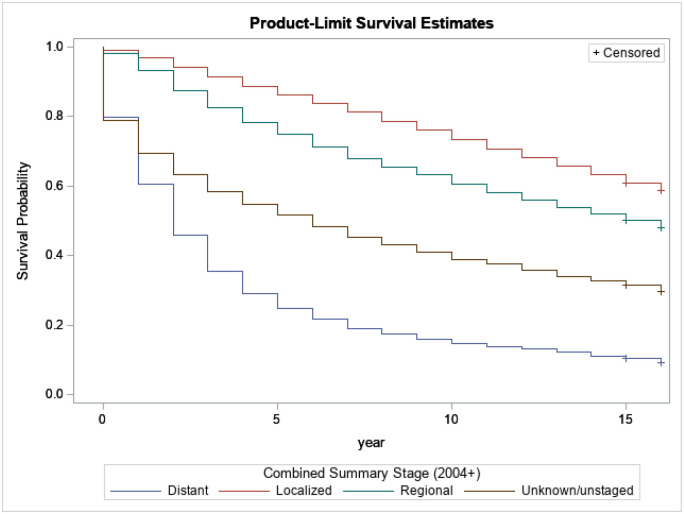
Kaplan-Meijer curves to compare survival of breast cancer patients by stage with a log-rank p-value<0.0001.

Figure 1 shows the relationship between survival probabilities for breast cancer patients and the different stages at diagnosis supported by the statistically significant log-rank test. We observe that the stage at diagnosis significantly plays a substantial role in the survival time of breast cancer patients. For instance, after 5 years of survival, only 9% of survivors with localized stages have died; however, at the same time, around 75% of survivors with distant stages have died. It is then crucial to promote and encourage early diagnosis to improve survival time among breast cancer patients.

**Figure 2:**
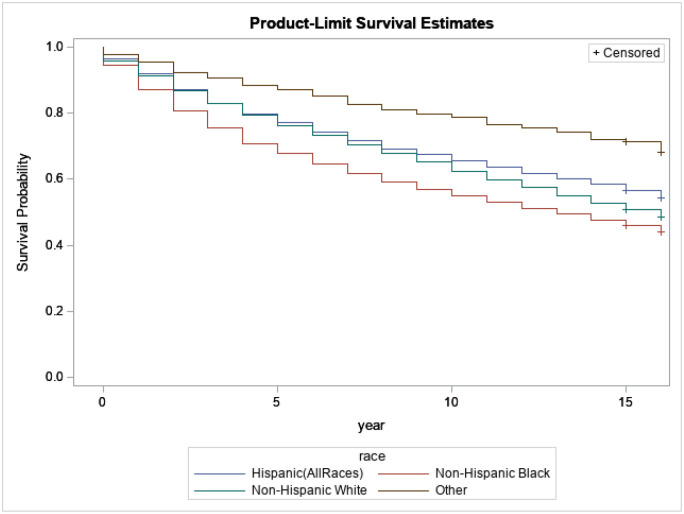
Kaplan-Meijer curves to compare survival of breast cancer patients by Race and ethnicity with a log-rank p-value<0.0001.

Figure 2 displays the difference in the survival probability of breast cancer patients among race and ethnicity groups. When performing the log-rank test, we notice a statistically significant disparity of survival probability based on race and ethnicity. For example, after 5 years of survival, around 30% of Black-non-Hispanics survivors died, around 21% of Hispanic survivors died, around 22% of White-non-Hispanics died, and only 10% of the other survivors died. Therefore, it is important to intensify the awareness of breast cancer within all communities, mainly among Black non-Hispanics.

**Figure 3:**
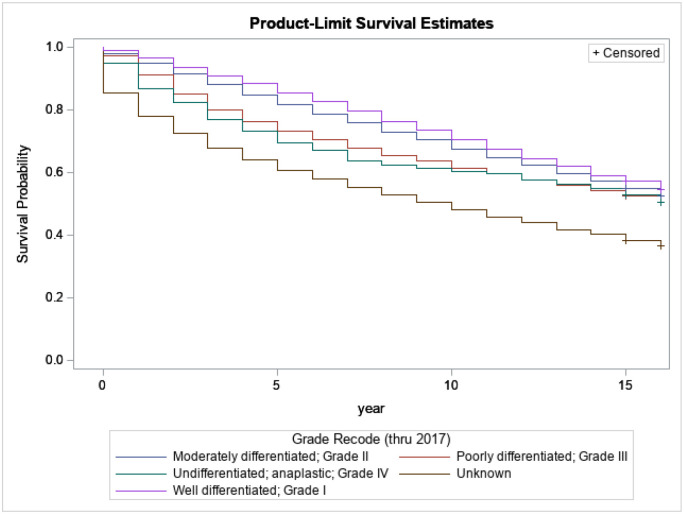
Kaplan-Meijer curves to compare survival of breast cancer patients by cancer grade at diagnosis with a log-rank p-value< 0.0001.

Figure 3 presents the relationship between survival probabilities for breast cancer patients and the different grades at diagnosis of breast cancer. We note that the grade at cancer diagnosis significantly influences patients’ survival time. For instance, after 5 years of survival, only 10% of survivors with grade I died. Meanwhile, at the same time, around 38% of survivors with grade 4 at diagnosis died, and 50% of them died by the 10th year. It is crucial to promote and encourage early diagnosis to improve survival time among breast cancer patients.

**Figure 4:**
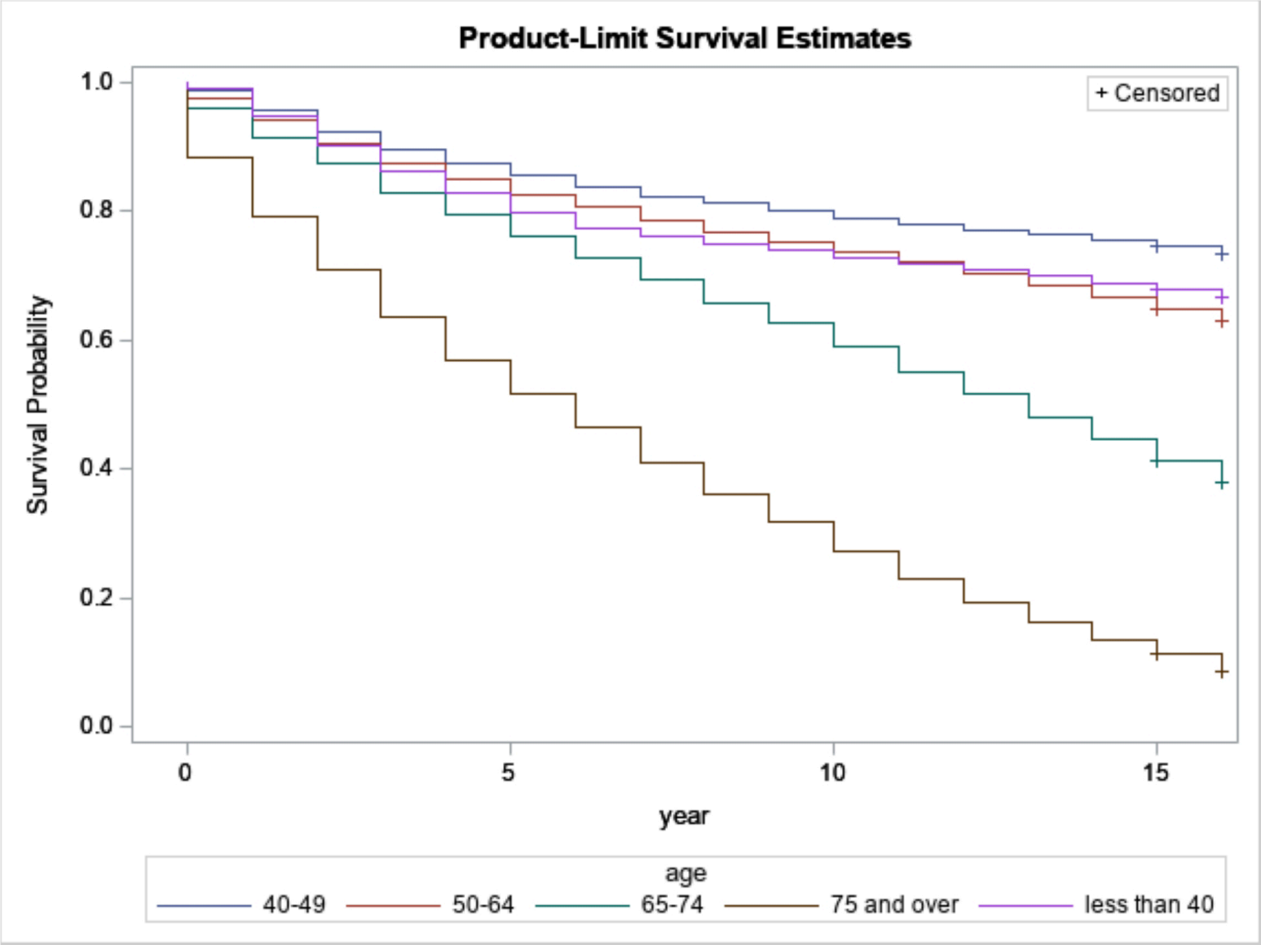
Kaplan-Meijer curves to compare survival of breast cancer patients by age at diagnosis with a log-rank p-value< 0.0001.

Figure 4 presents the relation between survival probabilities for breast cancer patients with regard to age. Based on age, survivors who were diagnosed at 75 years of age and over have the lowest survival time compared to the youngest. However, we know that as people get older, the probability of dying is increasing. Therefore, we need to be cautious when attributing the survival of patients who are 75 years or older to breast cancer only.

**Figure 5:**
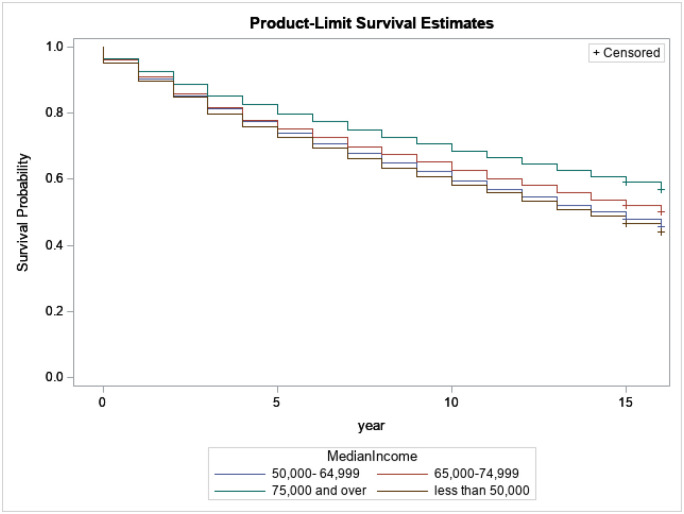
Kaplan-Meijer curves to compare survival of breast cancer patients by Median Household Income with a log-rank p-value<0.0001.

Figure 5 presents the relation between survival probabilities for breast cancer patients with regard to the median household income of the survivors at diagnosis. We note that survivors from households with a median income of $75,000.00 or over have a better survival length than other income categories. We should then better assist and help survivors from low-income households to improve their survival time.

**Figure 6:**
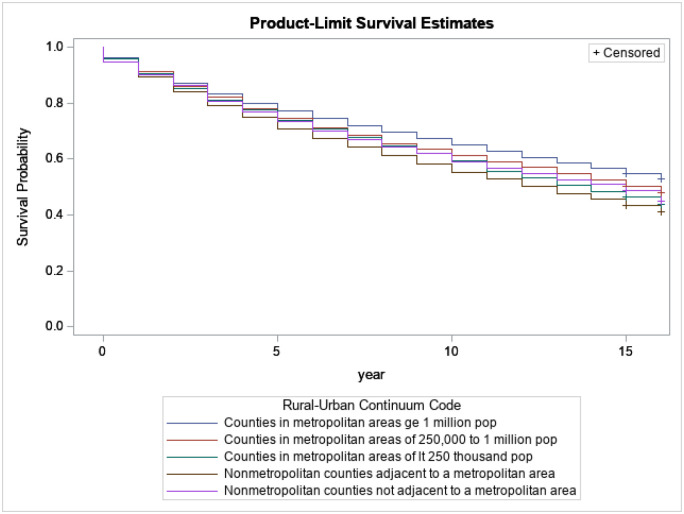
Kaplan-Meijer curves to compare survival of breast cancer patients by Areas where survivors lived at diagnosis with a log-rank p-value<0.0001.

Figure 6 presents the relation between survival probabilities for breast cancer patients with regard to areas where survivors lived at diagnosis. Based on the regions where survivors lived, survivors from counties in metropolitan areas greater than 1 million have a better survival length than survivors from smaller cities.

**Figure 7:**
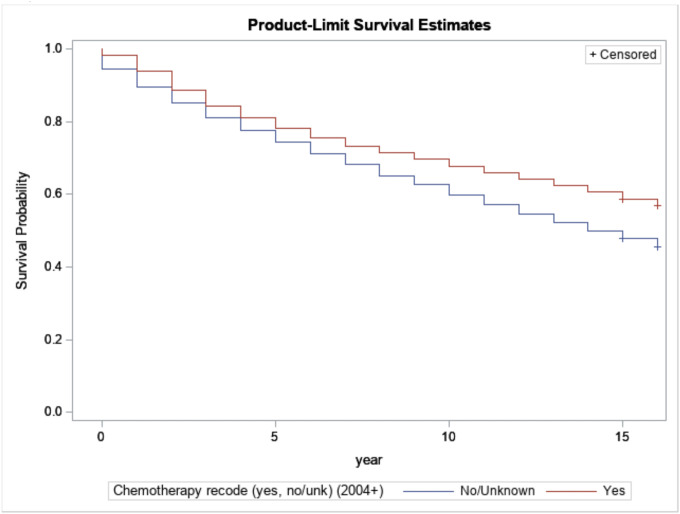
Kaplan-Meijer curves to compare survival of breast cancer patients by having chemotherapy or not with a log-rank p-value<0.0001.

Figure 7 compares the survival of the cancer survivors who have and did not have chemotherapy. Survivors who had chemotherapy have a statistically significant, more extended survival period.

**Figure 8:**
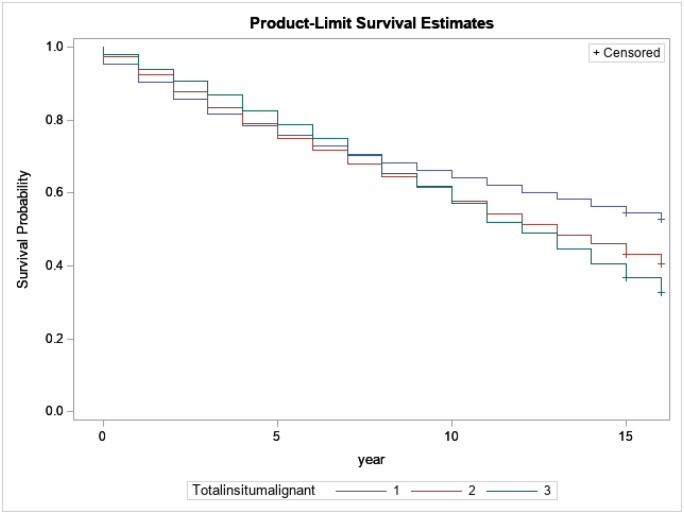
Kaplan-Meijer curves to compare survival of breast cancer patients by the number of cancers each survivor had at diagnosis with a log-rank p-value<0.0001.

Figure 8 presents the relation between survival probabilities for breast cancer patients with regard to the number of cancers each survivor has at diagnosis. Based on the number of cancers each survivor had, survivors with only one cancer have longer survival lengths compared to survivors with 2 or more.

**Figure 9:**
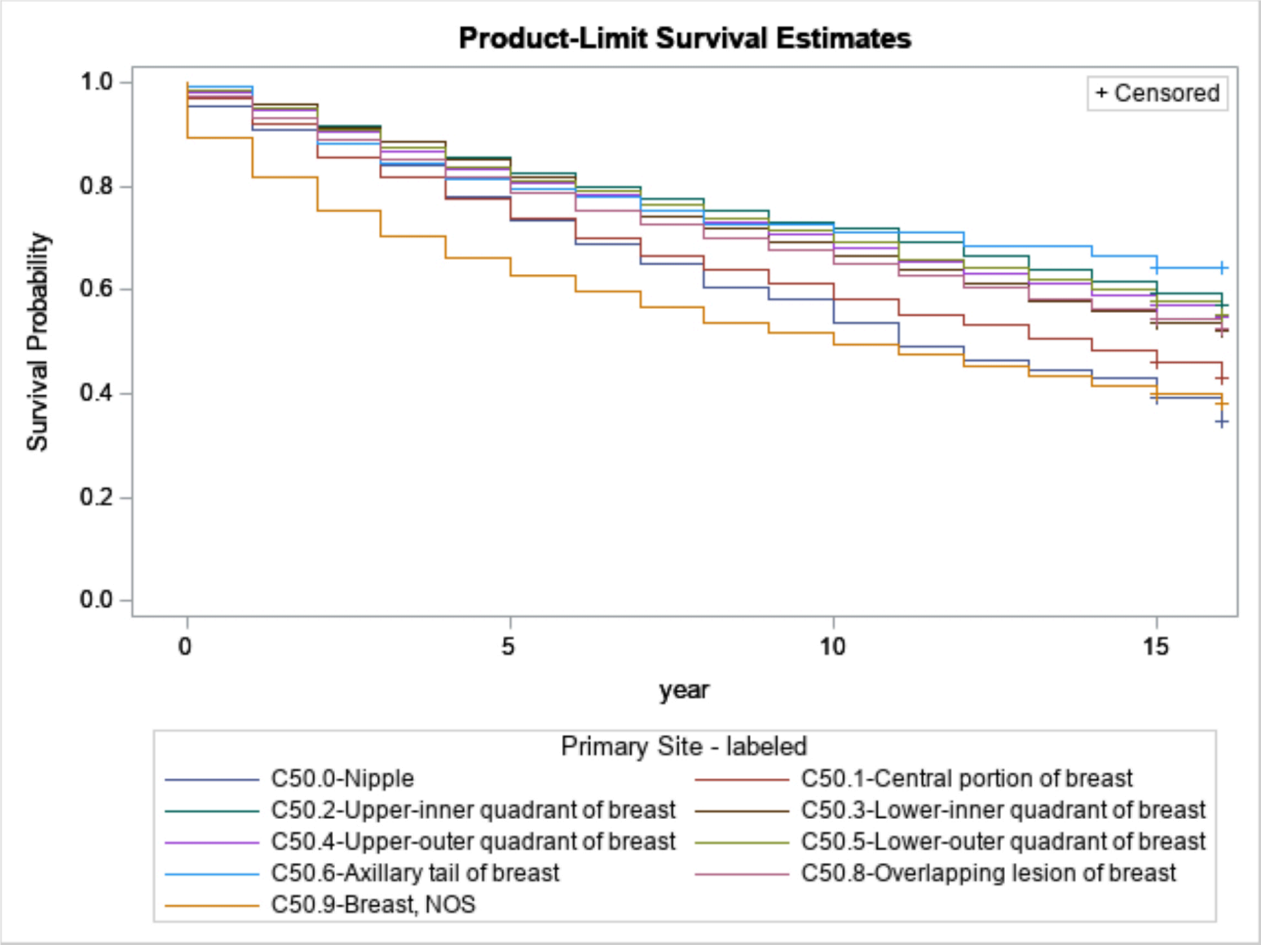
Kaplan-Meijer curves to compare breast cancer patients’ survival based on the breast cancer’s primary site with a log-rank p-value<0.0001.

Figure 9 presents the relationship between survival probabilities for breast cancer patients with regard to the primary site of breast cancer. Based on the primary site of the breast cancer, each survivor had survivors that had breast NOS as their primary site and had the lowest survival length. In contrast, survivors with the axillary tail of the breast had a more extended survival period.

Next, we performed a survival time regression modeling. We used a log-link function to measure the length of the survival period.

### Model 1: Survival Time Regression Model


$$\text{log}\left(s_{i}\right)=\beta_{0}+\beta_{1}{\text{Gender}}_{i}+\beta_{2}{\text{Race}}_{i}+\beta_{3}{\text{Age}}_{i}+\beta_{4}{\text{chemotherapy}}_{i}+\beta_{5}{\text{Grade}}_{i}+\beta_{6}{\text{Stage}}_{i}+\beta_{7}{\text{chemotherapy}}_{i}+\beta_{8}{\text{NumerofCancer}}_{i}+\beta_{9}{\text{Median HHIncome}}_{i}+\beta_{10}{\text{Geographic area}}_{i}+\epsilon_{i}\;\text{Where}\;\epsilon_{i}\sim N\left(0,\sigma^{2}\right),\text{and}\;s_{i}\;\text{represent survival time for each individual for}\;i=1,\cdots ,n\text{.}$$

The results of the survival time regression analysis are given in Table 3.

Compared to breast cancer patients who are Hispanic of all races, Black no-Hispanic has a shorter survival length (−0.2281**), White no-Hispanic has a slightly shorter survival length (−0.0212), but not statistically significant, and other survivors (0.2271**) has longer survival length.

Compared to patients under 40, survivors 50 or over have a shorter survival period, and patients between 40 and 49 years old have a more extended survival period. Compared to survivors diagnosed with grade IV, survivors with grades I or II have a more extended survival period. Compared to survivors with distant stages at diagnosis, survivors with Localized (1.4544**), regional (1.0448**) Unknown/Un-staged survivors (0.9431**) are living longer.

Compared to survivors from median household incomes of between $50,000.00 and $64,999.00, survivors from households with a median income of 75,000.00 or over live much longer.

Compared to survivors with the nipple as a primary site, survivors with the axillary tail of the breast are living longer.

Compared to survivors who had three or more cancers, survivors with one or two cancers are living longer.

We have also performed Cox Proportional Hazard regression of the data.

### Model 2: Cox Proportional Hazard Regression


$$h_{i}(t)=h_{0}(t)\;\text{exp}(\alpha_{1}{\text{Gender}}_{i}+\alpha_{2}{\text{Race}}_{i}+\alpha_{3}{\text{Age}}_{i}+\alpha_{4}{\text{chemotherapy}}_{i}+\alpha_{5}{\text{Grade}}_{i}+\alpha_{6}{\text{Stage}}_{i}+\alpha_{7}{\text{Median HHIncome}}_{i}+\alpha_{8}{\text{Geographic area}}_{i}+\alpha_{9}{\text{NumerofCancer}}_{i})$$

The results of the Cox Proportional Hazard regression are shown in Table 4.

From Table 4, compared to whites with breast cancer, blacks present a higher hazard ratio (1.282**), which means that blacks with breast cancer have 28.2% more risk of dying. Furthermore, compared to survivors aged between 0 and 39, survivors 75 and over present a higher hazard ratio (5.585**). Compared to survivors diagnosed with grade I, survivors with more advanced grades are more likely to die early; for instance, grade IV has a hazard ratio (1.429**), which means grade IV has 42.9% more risk of dying. Moreover, compared to survivors diagnosed with Localized stage breast cancer, survivors with more advanced stage are more likely to die early; for example, survivors with distant stage have a hazard ratio (5.947**) that means that at any particular time, survivors diagnosed with distant stage have 494.7% more risk of dying compared to those diagnosed with Localized Stage. We then need to promote early diagnosis to improve survival length.

Compared to survivors with a median household income of less than 50,000, survivors with a median household income of 75,000 or over have a statistically significant lower hazard ratio (0.920**).

Compared to survivors who have one cancer, survivors with multiple cancers are more likely to die early. For example, survivors with 2 cancers have a hazard ratio (1.233**), and survivors with three or more have a hazard ratio (1.360**) compared to survivors with one cancer.

Compared to survivors with the upper outer quadrant of the breast as a primary site, survivors with the axillary tail of the breast are living longer with a hazard ratio of 0.705**, and survivors with overlapping lesions of breast have a hazard ratio of 1.060* and survivors with breast, NOS have a hazard ratio of 1.251**. This indicates that survivors with overlapping lesions of the breast and survivors with breast NOS are more likely to die early compared to survivors with the upper outer quadrant of breast.

Therefore, based on those regressions, the most significant factors are the stage of diagnosis, age, grade, income, primary site, total number of cancers each patient has, and race.

Based on Table 5, there are more Hispanics that are alive at last contact (55.44%) than White non-Hispanics (49.55%) and Black non-Hispanics (45.11%). This supports our previous results that Hispanics are more likely to live longer than White and Black non-Hispanics.

From Table 6, based on the estimate, the most relevant results indicate that The best prognostic is a woman aged between 40 to 49 years old, diagnosed with localized stage and grade one with the Axillary tail of the breast as a primary site and with only one cancer. This is true for White non-Hispanics, Hispanics (all races), and Black non-Hispanics.

The worse prognostic is a woman aged 75 and over, diagnosed with distance stage and grade four with breast, not otherwise specified as a primary site and with only one cancer. This is true for White non-Hispanics, Hispanics (all races), and Black non-Hispanics. However, there are differences in the value of hazard ratios. For instance, Compared to White non-Hispanics aged less than 40, White non-Hispanics aged 75 or over have a hazard ratio of 6.808. Compared to Hispanics aged less than 40, Hispanics aged 75 or over have a hazard ratio of 4.438. Compared to Black non-Hispanics aged less than 40, Black non-Hispanics aged 75 or over have a hazard ratio of 4.028.

Compared to White non-Hispanics diagnosed with localized stage, White non-Hispanics diagnosed with distant stage have a hazard ratio of 5.760. Compared to Hispanics diagnosed with localized stage, Hispanics diagnosed with distant stage have a hazard ratio of 6.263. Compared to Black non-Hispanics diagnosed with localized stage, blacks diagnosed with distant stage have a hazard ratio of 6.648.

Based on the Chi-Square test, there is a strong correlation between the year of death and the histology recorded by broad groupings from SEER.

The three Histology that leads to a shorter survival length are: 8550–8559: acinar cell neoplasms, 9180–9249: osseous and chondromatous neoplasms, 8000–8009: unspecified neoplasm, 8010–8049: epithelial neoplasms, NOS, and 9120–9169: blood vessel tumors.

The three Histology that leads to a longer survival length are: 9580–9589: granular cell tumors & alveolar soft part sarcoma, 8810–8839: fibromatous neoplasms, 8590–8679: specialized gonadal neoplasms, 9000–9039: fibroepithelial neoplasms and 8560–8579: complex epithelial neoplasms.

Therefore, it is important to pay attention to the histology of breast cancer patients and continue working on finding better treatments for the most vulnerable patients.

## Conclusion

This study allows us to investigate the main factors that can explain the survival length of breast cancer patients in Texas from a 16-year retrospective cohort data. Our analysis shows that stage, grade, primary site of the cancer, number of cancers each patient has, histology of the cancer, age, race, and income are among the main variables that explain why some breast cancer survivors live much longer than others. For instance, compared to White non-Hispanics, Black non-Hispanics have a shorter length of survival with a hazard ratio of (1.282**). Compared to survivors aged between 0 and 39, survivors 75 and over present a higher hazard ratio (5.585**). Moreover, compared to survivors’ diagnoses with Localized stage breast cancer, survivors with distant stage have a hazard ratio (5.947**). Finally, compared to survivors diagnosed with grade I, survivors with grade IV have a hazard ratio (1.429**).

Hispanics have a better prognostic than White non-Hispanics and Black non-Hispanics. This may be explained by the fact that Hispanics are being diagnosed at a younger age than Black and White non-Hispanics and by family and strong social support that the Hispanic ethnicity group is known for. In addition, religion plays a crucial role in Hispanic communities as well compared to others. All of that may lead to more resiliency and a better outcome for Hispanic breast cancer survivors.

The best prognostic for White non-Hispanics, Hispanics (all races), and Black non-Hispanics is a woman aged between 40 to 49 years old, diagnosed with localized stage and grade one with Axillary tail of breast as a primary site with only one cancer and with a household income of 75,000.00 and over.

The worse prognostic is a woman aged 75 and over, diagnosed with distance stage and grade four with breast, not otherwise specified as a primary site and with only one cancer. This is true for White non-Hispanics, Hispanics (all races), and Black non-Hispanics.

Policymakers need to screen and diagnose patients early and better assist older people experiencing poverty to improve survival time since survival length has huge socio-economic implications.

Finally, clinicians are still working to find better treatment regimens for breast cancer survivors, and they need more resources. It is then crucial that cancer research continue to be a priority for our society. However, this paper has some limitations; we believe this study would have been more robust if we had had access to more survivors’ characteristics, such as education-level information and survivors’ comorbidities.

## Figures and Tables

**Table 1:** Exploratory descriptive analysis of the characteristics of breast cancer survivors who were diagnosed in 2004 and 2005 in Texas.

Variables	Number	White non-Hispanic	Black non-Hispanic	Hispanic (all race)	Other
**Race**					
White non-Hispanic	17033	17033			
Black non-Hispanic	2895		2895		
Hispanic (all Race)	4690			4690	
Other	566				566
**Age**					
between 0 and 39	1515	4.02%	9.67%	10.30%	12.01%
between 40 and 49	4707	15.97%	22.25%	24.78%	31.80%
between 50 and 64	9143	36.23%	36.89%	35.95%	38.52%
between 65 and 74	4842	20.77%	16.48%	16.14%	12.37%
Over 75	4977	23.01%	14.72%	12.84%	5.30%
**Gender**					
Female	24992	99.16%	98.96%	99.66%	99.47%
Male	192	0.84%	0.34%	0.34%	0.53%
**Treatment**					
Chemotherapy (yes)	8904	31.70%	41.90%	43.84%	41.52%
Chemotherapy (No)	16280	68.30%	58.10%	56.16%	58.48%
**Total in situ\ malignant**					
Total in situ \malignant = 1	19338	75.30%	79.17%	79.68%	85.51%
Total in situ \malignant = 2	4967	20.83%	17.72%	17.83%	12.37%
Total in situ\ malignant ≥3	879	3.87%	3.11%	2.49%	2.12%
**Grade**					
Grade I	3540	16.06%	8.01%	10.70%	12.37%
Grade II	8194	34.38%	25.87%	30.19%	30.57%
Grade III	8923	32.50%	45.91%	39.70%	34.63%
Grade IV	519	1.71%	2.63%	2.99%	2.12%
Unknown grade	4008	15.35%	17.58%	16.42%	20.32%
**Stage**					
Localized	13091	55.36%	43.07%	45.42%	50.18%
Regional	8338	31.02%	37.44%	38.08%	32.69%
**Race**					
Distant	1498	5.31%	8.57%	6.84%	4.24%
Unknown/Un-staged	2257	8.31%	10.92%	9.66%	12.90%
**Primary site of breast cancer**					
Nipple	256	1.08%	0.52%	1.17%	0.35%
Central portion of breast	1077	4.37%	3.45%	4.48%	3.89%
Upper-inner quadrant of breast	2415	9.51%	10.26%	9.32%	10.95%
Lower-inner quadrant of breast	1155	4.64%	4.77%	4.33%	4.24%
Upper outer quadrant of breast	7594	31.20%	27.67%	28.19%	27.56%
Lower outer quadrant of breast	1575	6.48%	5.84%	5.93%	4.42%
Axillary tail of breast	146	0.52%	0.86%	0.60%	0.88%
Overlapping lesion of breast	4620	18.53%	18.58%	17.42%	19.26%
Breast, not otherwise specified (NOS)	6346	23.68%	28.05%	28.57%	28.45%
**Median HH Income**					
Less than 50,000	2819	8.81%	4.94%	24.80%	2.12%
50,000–64,999	8543	34.27%	25.28%	40.09%	16.61%
65,000–74,000	7731	28.93%	50.54%	23.07%	45.76%
75,000 and over	6091	27.99%	19.24%	12.05%	35.51%
**Areas where survivors lived**					
Metropolitan areas pop > 1million	15703	62.67%	74.72%	50.72%	86.04%
Urban areas	2779	7.26%	5.42%	28.96%	4.95%
250k < pop < 1million					
Urban areas	3058	13.37%	10.54%	9.51%	5.30%
Pop = 250k					
Rural areas	2668	12.40%	7.67%	6.78%	2.83%
Adjacent to a metropolitan					
Rural areas	976	4.31%	1.66%	4.03%	0.88%
Not Adjacent to a metropolitan					

**Table 2:** Summary of the Chi-square test between race and the other variables.

Variable	Chi-square test statistic	Degrees of Freedom (DF)	P-Value
Stage	281.7296	9	< 0.0001
Grade	434.0589	12	< 0.0001
Primary site of the tumor	103.9056	24	< 0.0001
Age	958.9285	12	< 0.0001
Gender	15.6139	3	< 0.0014
Chemotherapy(yes/No)	310.7177	3	< 0.0001
Median Household Income	2137.6286	9	< 0.0001
Rural-Urban Code	2217.3659	12	< 0.0001
Total in situ \malignant for each patient	83.1574	6	< 0.0001

**Table 3:** Survival Time Regression Analysis.

Parameter		Estimate
**Intercept**		1.9547**
**Gender**	Reference for gender: Male	
	Female	0.2280**
**Race**	Reference for Race: Hispanic (all races)	
	Other	0.2217**
	White no-Hispanic	−0.0212
	Black no-Hispanic	−0.2281**
**Age**	Reference for age: less than 40	
	Between 40 and 49	0.1963**
	Between 50 and 64	−0.1226**
	between 65 and 74	−0.6881**
	over 75	−1.4106**
**Treatment**	Reference for Treatment: Chemotherapy (Yes)	
	Chemotherapy (No/Unknown)	0.0176
**Grade**	Reference for Grade: Grade IV	
	Grade I	0.2497**
	Grade II	0.1712**
	Grade III	−0.0046
	Unknown	0.0863
**Stage**	Reference for stage: Distant	
	Localized	1.4544**
	Regional	1.0448**
	Unknown/Un-staged	0.9431**
**The primary site of breast cancer**	Reference for Primary site: Nipple	
	Central portion of breast	−0.0971
	Upper-inner quadrant of breast	0.0148
	Lower-inner quadrant of breast	−0.0081
	Upper-outer quadrant of breast	0.0291
	Lower outer quadrant of breast	0.0518
	Axillary tail of breast	0.3246**
	Overlapping lesion of breast	−0.0156
	Breast, NOS	−0.1288
**Median HH income**	Reference for median household income: 75,000 and over	
	Less than 50,000	−0.0680
	50,000–64,999	−0.0724**
	65,000–74,999	−0.0702**
**Areas where survivors lived**	Reference for Areas where survivors lived: Rural areas not adjacent to a metropolitan	
	Metropolitan area of over 1 million people	−0.0713
	Population of 250,000 to 1 million	−0.0382
	Population of 250,000	−0.1377**
	Rural area adjacent to a metropolitan	−0.1471**
**Total in situ\ malignant**	Reference for total in situ\malignant: Total in situ\ malignant ≥ 3	
	Total in situ\ malignant = 1	0.2967**
	Total in situ\ malignant = 2	0.0909**

Note:

** means statistically significant at 5% level (p-values are less than 5%)

**Table 4:** Cox proportional hazards regression model analysis.

Parameter		Estimate	Hazard Ratio
**Gender**	Reference for gender: Male		
	Female	−0.23053**	0.794
**Race**	Reference for Race: White non-Hispanics		
	Hispanics (all races)	−0.02885	0.972
	Black non- Hispanics	0.24881**	1.282
	Other	−0.29233**	0.747
**Age**	Reference for age: Less than 40		
	Between 40 and 49	−0.19927**	0.819
	Between 50 and 64	0.19718**	1.218
	between 65 and 74	0.86458**	2.374
	over 75	1.72016**	5.585
**Treatment**	Reference for Treatment: Chemotherapy (No/Unknown)		
	Chemotherapy (yes)	−0.00852	0.992
**Grade**	Reference for grade: grade I		
	Grade II	0.10576**	1.112
	Grade III	0.32166**	1.379
	Grade IV	0.35707**	1.429
	Unknown grade	0.27725**	1.319
**Stage**	Reference for stage: Localized		
	Regional	0.49950**	1.648
	Distant	1.78286**	5.947
	Unknown/ Un-staged	0.81844**	2.267
**Primary site of the breast cancer**	Reference for primary site:		
	Upper-inner quadrant of breast		
	Nipple	0.04785	1.049
	Central portion of breast	0.14846**	1.160
	Upper-inner quadrant of breast	0.00558	1.006
	Lower-inner quadrant of breast	0.04966	1.051
	Lower outer quadrant of breast	−0.02984	0.971
	Axillary tail of breast	−0.34928**	0.705
	Overlapping lesion of breast	0.05791**	1.060
	Breast, NOS	0.22414**	1.251
**Median HH income**	Reference for median household income: less than 50,000		
	50,000 to 64,999	0.01499	1.015
	65,000 to 74,999	−0.01326	0.987
	75,000 and over	−0.08324	0.920
**Areas where survivors lived**	Reference for Areas where survivors lived: Metropolitan area over 1 million people		
	Metropolitan area, Population of 250,000 to 1 million	−0.06848	0.987
	Metropolitan areas, Pop = 250,000	0.05341	1.055
	Nonmetropolitan counties adjacent to a metropolitan.	0.06580	1.068
	Nonmetropolitan counties not adjacent to a metropolitan.	−0.09393	0.910
**Total in situ\ malignant**	Reference for total in situ\malignant: Total in situ\ malignant	0.20963**	1.233
	=1	0.30716**	1.360
	Total in situ\malignant = 2		
	Total in situ \malignant ≥3		

Note:

** means 5% level of statistical significance (p-values are less than 5%)

**Table 5:** Summary of the Number of Events and Censored

Race and Ethnicity	Total number	Event (died)	Censored (Alive at last contact)	Percent Censored
Hispanics	4690	2090	2600	55.44%
White no-Hispanics	17033	8574	8459	49.55%
Black no-Hispanics	2895	1589	1306	45.11%

**Table 6:** **Cox proportional hazards regression model estimate for** Hispanics, White non-Hispanics, and Black non-Hispanics.

Parameter		White non-Hispanic		Hispanic (all Races)		Black non-Hispanic	
		Estimate	Hazard Ratio	Estimate	Hazard Ratio	Estimate	Hazard Ratio
**Gender**	female	−0.2019**	0.817	−0.2299	0.795	−0.29661	0.743
**Age**	Between 40 and 49	−0.17802**	0.837	−0.25012**	0.779	−0.01393	0.986
	Between 50 and 64	0.29121**	1.338	0.13038	1.139	0.24490**	1.277
	between 65 and 74	1.01684**	2.764	0.68519**	1.984	0.73323**	2.082
	over 75	1.91803**	6.808	1.49029**	4.438	1.39337**	4.028
**Treatment**	Chemotherapy (No/Unknown)	−0.00379	0.996	0.01425	1.014	−0.06379	0.938
**Grade**	Grade II	0.11478**	1.122	0.10424	1.110	0.10978	1.116
	Grade III	0.33043**	1.392	0.32544**	1.341	0.33218**	1.394
	Grade IV	0.41753**	1.518	0.29305**	1.341	0.28613	1.331
	Unknown	0.29362**	1.341	0.27436**	1.316	0.24639**	1.279
**Stage**	Regional	0.46051**	1.585	0.58007**	1.786	0.61182**	1.844
	Distant	1.75091**	5.760	1.83458**	6.263	1.89431**	6.648
	Unknown/Un-staged	0.80528**	2.237	0.70091**	2.016	1.08207**	2.951
**Primary site of the breast cancer**	Nipple	0.08389	1.088	−0.07164	0.931	0.18040	1.198
	Central portion of breast	0.15491**	1.168	0.07509	1.078	0.33149**	1.393
	Upper-inner quadrant of breast	0.00865	1.009	−0.05039	0.951	0.03463	1.035
	Lower-inner quadrant of breast	0.05101	1.052	0.04518	1.046	−0.01716	0.983
	Lower outer quadrant of breast	0.000799	1.001	−0.11491	0.891	−0.07617	0.927
	Axillary tail of breast	−0.29783	0.742	−0.39051	0.677	−0.40060	0.670
	Overlapping lesion of breast	0.02242	1.023	0.11109	1.117	0.13943	1.150
	Breast, NOS	0.20422**	1.227	0.27699**	1.319**	0.22052**	1.247
**Median HH income**	50,000–64,999	0.02350	1.024	0.03806	1.039	0.08644	1.090
	65,000–74,999	0.00148	1.001	−0.04210	0.959	0.06234	1.064
	75,000 and over	−0.04818	0.953	−0.20555	0.814	−0.03269	0.968
**Areas where survivors lived**	Population of 250,000 to 1 million	−0.11037**	0.895	−0.00765	0.992	−0.07764	0.925
	Population of 250,000	0.04848	1.050	0.15052	1.162	−0.00624	0.994
	Rural area adjacent to a metropolitan	0.06419	1.066	0.14872	1.160	−0.00796	0.992
	Rural area not adjacent to a metropolitan	−0.11180	0.894	0.04773	0.7155	−0.23138	0.793
**Total in situ\ malignant**	**Total in situ\ malignant** = 2	0.17584**	1.192	0.31111**	1.365	0.20792**	1.231
	**Total in situ\ malignant** ≥ 3	0.27750**	1.320	0.43392**	1.543	0.33712**	1.401

Note:

** means 5% level of statistical significance (p-values are less than 5%)

**Table 7:** Histology recodes broad grouping by SEER.

Histology recodes broad grouping by SEER	Frequency	Percent of survivors that died in 5\varvec*y*\varvec*e*\varvec*a*\varvec*r*\varvec*s* or less	Percentageof survivors who died between 5 and 16 years.	Percentage of survivors that are still alive
**8000–8009: unspecified neoplasm**	1042	59.4%	14.78%	25.82%
**8010–8049: epithelial neoplasms, NOS**	763	46.92%	21.76%	31.32%
**8050–8089: squamous cell neoplasms**	93	27.96%	35.48%	36.56%
**8140–8389: adenomas and adenocarcinomas**	611	31.59%	24.55%	43.86%
**8390–8429: adnexal and skin appendage neoplasms**	26	26.92%	34.62%	38.46%
**8440–8499: cystic, mucinous and serous neoplasms**	519	21.39%	37.95%	40.66%
**8500–8549: ductal and lobular neoplasms**	21912	21.66%	25.34%	53%
**8550–8559: acinar cell neoplasms**	1	100%	0%	0%
**8560–8579: complex epithelial neoplasms**	107	35.51%	18.7%	45.79%
**8590–8679: specialized gonadal neoplasms**	1	0%	0%	100%
**8800–8809: soft tissue tumors and sarcomas, NOS**	12	50%	16.67%	33.33%
**8810–8839: fibromatous neoplasms**	4	0%	0%	100%
**8890–8929: myomatous neoplasms**	2	50%	0%	50%
**8930–8999: complex mixed and stromal neoplasms**	10	40%	50%	10%
**9000–9039: fibroepithelial neoplasms**	68	17.65%	8.82%	73.53%
**9120–9169: blood vessel tumors**	9	55.55%	11.12%	33.33%
**9180–9249: osseous and chondromatous neoplasms**	3	66.67%	0%	33.33%
**9580–9589: granular cell tumors & alveolar soft part sarcoma**	1	0%	0%	100%
**Total**	25184	24.34%	24.99%	50.67

Statistics for Table of Histologyrecodebroadgrouping by Year_of_death_recode

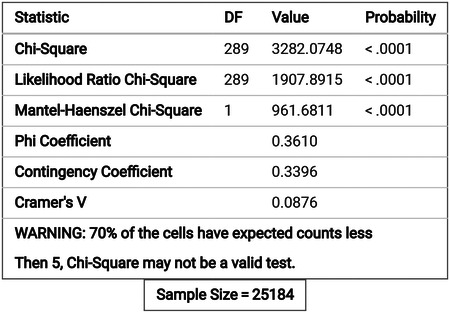

## Data Availability

*The datasets generated during and/or analyzed during the current study was* provided by the Surveillance, Epidemiology, and End Results (SEER) Program (www.seer.cancer.gov) SEER*Stat Database: Incidence - SEER Research Plus Limited-Field Data, 22 Registries, Nov 2022 Sub (2000–2020) - Linked to County Attributes - Time Dependent (1990–2021) Income/Rurality, 1969–2021 Counties, National Cancer Institute, DCCPS, Surveillance Research Program, released April 2023, based on the November 2022 submission.
